# Toward model-guided electrophysiology—Encoding of chirps in the electrosensory periphery of *Apteronotus leptorhynchus*

**DOI:** 10.3389/fncom.2026.1827196

**Published:** 2026-05-29

**Authors:** Alexandra Barayeu, Jan Benda, Jan Grewe

**Affiliations:** 1 Neuroethology, Institute for Neurobiology, University of Tübingen, Tübingen, Germany; 2 Bernstein Center for Computational Neuroscience Tübingen, Tübingen, Germany

**Keywords:** communication, electrosensory, LIF-model, modeling, sensory encoding, chirps

## Abstract

Models formalize our understanding of a system and generate hypotheses that can be tested experimentally. In this study, we use a previously developed model of p-type electroreceptor afferents to support electrophysiological observations regarding the encoding of chirps in the electrosensory periphery of the weakly electric fish *Apteronotus leptorhynchus*. These animals employ their self-generated quasi-sinusoidal electric fields to navigate, find prey, and communicate. Electrocommunication happens in an electrosensory context that is defined by the superposition of the electric fields of the interacting animals. Within this context, chirps—brief excursions of the electric organ discharge frequency—interrupt the periodic interference pattern and are encoded in the responses of the electroreceptor afferents. Behavioral observations highlighted the immediate importance of chirps happening in contexts that were believed to be far outside the electroreceptor tuning, i.e., not encoded at all. Combining experiments with modeling, we show that chirps are nevertheless encoded under these conditions, identify how the encoding works in such contexts, and provide a deeper understanding of chirp encoding in the electrosensory periphery.

## 1 Introduction

Inter-individual communication is an important mechanism to convey information between animals or to announce one's presence or intent. For humans, vocal communication feels most natural but there are also other means such as olfactory or visual cues by which animals relay information. In electric fish, the ever-present electric organ discharge (EOD) is their way of gathering information about the outside world in their ecological niche in African or South-American freshwaters (Crampton, 2019) and also to communicate with conspecifics (Henninger et al., 2018). During communication, a relatively small set of seemingly stereotyped frequency modulations of their EOD are observed during animal interactions (Zupanc, 2002; Petzold et al., 2016). Despite being stereotyped frequency modulations, they are produced in various contexts and flavors. Furthermore, electrocommunication depends strongly on spatial distances and electrical properties of the surrounding waters (Benda, 2020). All these affect the electric signals that are processed by the electrosensory system and create a vast stimulus space. This space is much larger than can be realistically investigated using electrophysiological approaches. We thus tackle the problem of sensory encoding of communication signals in the electrosensory system with a combined experimental and modeling approach.

**Figure 1:**
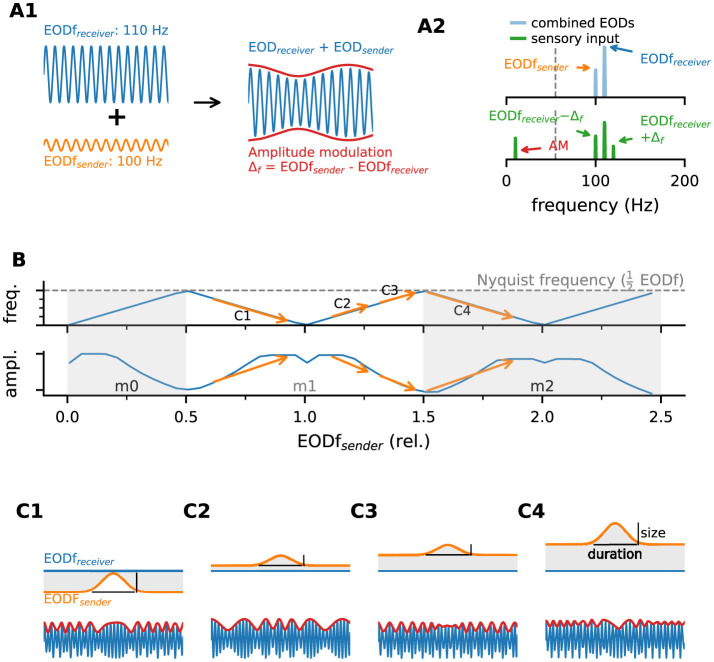
Amplitude modulations through interactions of EODs. **(A1)** When two wave-type electric fish interact, their EODs superimpose and each animal senses its self-generated field amplitude modulated by the other's EOD. From the perspective of the *receiver*, the own field has the stronger amplitude while the amplitude of the *sender* is attenuated by spatial distance. If the two EOD frequencies are in a similar range, the amplitude modulation (AM, red envelope) has a frequency that is given by the difference (Δ_*f*_) between the individual frequencies. **(A2)** The power spectrum of the combined EODs (blue) contains only two spectral components, a peak at EODf_*receiver*_ and at EODf_*sender*_. Non-linear processes in the sensory periphery generate additional spectral peaks flanking the receiver peak at a distance of ±Δ_*f*_ and a peak at the AM frequency arises. The gray vertical line indicates half of the *EODf*_*receiver*_ with which the animal samples the environment (Nyquist frequency). **(B)** Repetitive nature of the AM's frequency and amplitude. If the difference between *receiver* and *sender* *EODf* exceeds the Nyquist frequency of the *receiver*'s sampling frequency, the AM frequency folds over and even though Δ_*f*_ increases, the AM frequency decreases. The amplitude of the spectral AM peak shows a similar repetitive structure. This aliasing effect leads to repetitive tuning observed in P-units (for details see Barayeu et al., 2023). Sectors *m0 – m2* denote stimulus frequency ranges of the zeroth, first, and second multiple of the *receiver's* EODf. Orange arrows indicate the transient changes induced by the chirps emitted by the *sender* shown in **(C1–C4)**. The arrows start at the unperturbed beat frequency and end at the peak AM frequency during the chirps. Depending on the type of encounter, the resting AM frequency is different and chirps have very different effects (Walz et al., 2014). **(C1)** The *sender's* EODf is well below the *receiver's* EODf. For the duration of the chirp, the AM frequency and response amplitude is changed as indicated by the respective two orange arrows in B. In this example, the relatively high AM frequency and the corresponding low spectral amplitude changes toward a stronger but slower AM during the chirp (red envelope of *receiver's* EOD below) which will cause different responses in the electroreceptor afferents. **(C2–C4)** Same as **(C1)**, but for different beat frequencies, chirp sizes and chirp durations.

In wave-type electric fish which continuously discharge their electric organ, such as *Apteronotus leptorhynchus*, the frequency of discharge (EOD frequency) contains information about the sex of the animal as well as their hierarchical status (Meyer et al., 1987; Zakon and Dunlap, 1999; Triefenbach and Zakon, 2008; Fugère et al., 2011; Raab et al., 2021). When two animals interact, their quasi-sinusoidal electric fields superimpose and each animal will experience its own electric field being amplitude modulated by the presence of the other (Figure 1A). Periodic amplitude modulations (AMs) are also referred to as beats (Heiligenberg, 1989; Benda, 2020), where the beat amplitude reflects the relative strengths of the signals while the beat frequency depends on the EOD frequencies of the interacting animals. In *A. leptorhynchus*, males have higher EOD frequencies (700 ≤ *EOD*_*f*_ < 1, 000 Hz) than females (500 ≤ *EOD*_*f*_ < 700 Hz, Meyer et al., 1987). Because of this sexual dimorphism, same-sex encounters typically lead to low-frequency beats, while opposite-sex encounters induce higher beat frequencies (Henninger et al., 2018). The beat thus sets the electrosensory stage for electric communication and affects the animals' behavior (Bastian and Nguyenkim, 2001; Engler and Zupanc, 2001; Hupé and Lewis, 2008). For electric communication, the fish utilize frequency modulations (Hagedorn and Heiligenberg, 1985; Zupanc and Maler, 1993; Moortgat et al., 1998; Zakon et al., 2002; Raab et al., 2021). During a so-called chirp, the difference between the EOD frequencies transiently changes, which leads to discontinuities in the regular beat pattern (Figures 1B, C, see also Benda et al., 2005).

The tuberous, or active electrosensory system, is tuned to the EOD frequency of the fish (Hopkins, 1976; Viancour, 1979). The most abundant type of electroreceptor afferent, the so-called P-units, encodes AMs through modulations of their firing rate (e.g., Gussin et al., 2007; Grewe et al., 2017; Grewe, 2020; Sinz et al., 2020; Hladnik and Grewe, 2023). The response strength to pure beats decays with increasing beat frequency, and it was assumed that beats beyond approximately 250 Hz cannot be encoded in the electrosensory system (e.g., Walz et al., 2014). In response to the discontinuities induced by chirps, P-units show brief changes in their firing rate and/or the response synchronization that could be explained by shifts along the beat tuning curve (Benda et al., 2005, 2006; Walz et al., 2014). Behavioral observations in the animals' natural habitat revealed that the usually investigated range of beat frequencies below 250 Hz is much too narrow and that courtship behaviors happen at frequencies exceeding 400 Hz (Henninger et al., 2018, 2020). This raised the question of how the resulting electrosensory signals can be encoded at all.

Recent studies on the encoding of unperturbed beats revealed that frequency folding at the Nyquist frequency of the EOD carrier frequency with which the animals sample their environment enables P-units to encode the entire behaviorally relevant range of beat frequencies (Barayeu et al., 2023). In this study, we test whether predictions based on the resulting repetitive tuning hold and whether chirp encoding is also repetitive. Since the stimulus space is much more extensive than can be sampled experimentally, we use model simulations to predict chirp encoding over a wide range of stimulus parameters and use electrophysiological recordings to test the validity of our simulations in a narrower range.

## 2 Results

Within this study, we used experimental data gathered from 64 P-units recorded in 13 weakly electric fish of the species *Apteronotus leptorhynchus* (see methods). For more extensive scanning of the vast stimulus space, we performed model simulations using 32 model neurons that were fitted to previously recorded individual P-units (see methods, Equations 1 – 7). We then employed methods of signal-detection theory to characterize the encoding of chirps in a wider stimulus space and greater depth than would be feasible with electrophysiological experiments alone.

Chirp encoding depends strongly on a number of factors: (i) the difference between the EOD frequencies, (ii) the relative EOD amplitudes of sender and receiver, (iii) the chirp size, i.e., the height of the frequency excursion, (iv) the chirp duration, (v) the phase in the beat period in which the chirp was emitted, and (vi) the readout timescale: High temporal precision leads to the best chirp detection performance over a wider range of beat frequencies.

### 2.1 Chirps at high difference frequencies are encoded in real and model P-units

Electric fish of the species *A. leptorhynchus* discharge their electric organs (electric organ discharge, EOD) to surround themselves with an electric field. The discharge frequency (*EODf* ) is individual-specific and very stable over longer timescales. Furthermore, *A. leptorhynchus* show a sexual dimorphism in the sense that male fish tend to have higher frequencies than female fish. The superposition of the EODs of nearby animals produces amplitude modulations (AMs, red in Figures 1A, C), also called beat. P-type electroreceptor afferents respond to AMs and encode the AM in their spiking activity. Electrocommunication signals, so-called chirps, happen in front of the background AMs and transiently modulate the AM. The same chirp, however, can have very different effects on the AM (Figures 1B, C). Consequently, the neuronal responses to the same chirp depend on the electrosensory context, here with respect to the *EODf* s of the interacting animals, in which it happens. We investigated the encoding of chirps for a much larger range of AMs than has been done before. Previous work on beat encoding (Barayeu et al., 2023) shows that our P-unit model is well able to reproduce the encoding of AMs over a wide range of electrosensory contexts, i.e., the relation of the recorded fish's EOD frequency (*EODf* ) and that of the communication partner.

**Figure 2:**
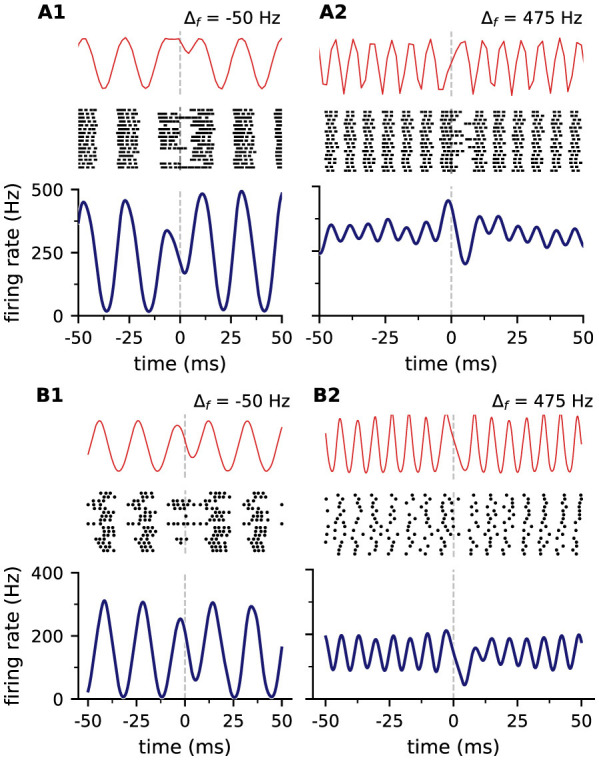
Comparison of experimental and model data. **(A)** Data recorded in cell *2019-10-28-ae-invivo-1* for two different stimulus conditions. The EOD frequency of the artificial communication partner is 50 Hz below or 475 Hz above that of the recorded fish (611 Hz, **A1** and **A2**, respectively). The chirp had a chirp size of 100 Hz and a duration of 14 ms. The strength of the sender fish's signal was calibrated to induce an AM of 20% of the recorded fish's EOD amplitude. The top graph shows the amplitude modulation extracted from the experimental data, and the time of the chirp's center is highlighted by the vertical dashed line. The center panel shows the cellular responses to 16 repetitions of the same chirp in a similar phase of the beat as spike rasters. The bottom panel depicts the firing rate estimated by kernel density estimation with a Gaussian kernel of 1.5 ms standard deviation. Time zero denotes the center of the chirp. **(B1, B2)** The same displays of simulated P-unit responses. Note that this is not a fit of the recorded cell shown in **(A)** but a model cell (2013-04-10-aa-invivo-1) chosen because of the similarity in *EODf* (623 Hz).

Figures 2A, B compares the responses of an actually recorded P-unit to responses of a model cell. During a chirp at a relatively small negative frequency difference between the receiving and the sending fish (Δ_*f*_ = −50 Hz), the sender's *EODf* transiently exceeds that of the receiver and the otherwise periodic AM is interrupted by the chirp. The discontinuity in the AM is reflected in the pattern of action potentials fired by the recorded P-unit. The firing rate (bottom plots in Figure 2) in response to the beat shows a very strong modulation ranging from almost 500 Hz during the peak of the AM down to a complete cessation of firing in the trough of the AM. The AM frequency matches the absolute of Δ_*f*_ (50 Hz). During the chirp, the rate modulation is less strong and the across-trial correlation is reduced which is consistent with previous work (Benda et al., 2005, 2006; Walz et al., 2014). Previous work on chirp coding assumed that difference frequencies beyond 250 Hz are outside the tuning of the P-units and chirp encoding at higher frequencies was never studied. Previous studies did not consider the frequency-folding effect at the Nyquist frequency of the receiver's *EOD*_*f*_. Frequency folding, however, folds the arising AM frequencies back toward lower frequencies and possibly back into the beat tuning of the cells. The *EODf* of the recorded animal shown in Figure 2A was at 611 Hz, and thus the Nyquist frequency was 305.5 Hz (*EODf*/2). In the experiment shown in Figure 2A1, the artificial sender's *EODf* was 561 Hz (50 Hz below the receiver's *EODf* ) which induced an AM frequency of 50 Hz which could be well sampled by the receiver as it was below the Nyquist frequency and there was no frequency folding. The sender's *EODf* in Figure 2A2, however, was 475 Hz above the receiver's *EODf* , and the Δ_*f*_ of 475 Hz clearly exceeded the receiver's Nyquist frequency (305.5 Hz). Accordingly, frequency folding occurred and led to an AM of 136 Hz. This folded frequency was well within the beat tuning and hence well encoded by the recorded P-unit. The firing rate modulation (bottom plot) was less pronounced than at −50 Hz (Figure 2A1), but the firing rate during the unperturbed AM (before and after the chirp) nevertheless reflects the AM. During the chirp (chirp size of 100 Hz), Δ_*f*_ increased further (toward 575 Hz at the chrip peak) but frequency folding led to an AM frequency reduction to 36 Hz to which P-units respond strongly. Accordingly, the rate modulation during the chirp exceeds that during the beat which was completely unexpected from previous understanding.

The P-unit models reproduced these observations; Figures 2B1, B2 show the model responses to the same stimulus conditions as were experimentally applied. The model cell, however, was not a fit of the recorded cell but one model from our list of models, chosen because it is based on data originating from an animal with a similar *EODf*. In this model cell, the overall firing rate was slightly lower than in the experimental data but the response patterns during the unperturbed beat and during the chirp are qualitatively the same.

### 2.2 Repetitive tuning of chirp encoding in experiment and model

To characterize chirp encoding, we analyzed how well neuronal responses to chirps can be distinguished from responses to the beat. For this, 20 ms segments that were centered on the chirp time of chirp- and beat-responses with similar phases were cut out of the recorded/simulated neuronal responses. A ROC analysis was performed on the spike train distances among the beat responses, the null distribution, and the distances between chirp- and beat-responses, the test distribution (see Methods). The fundamental assumption here was that a chirp is encoded in the responses if the chirp response is more different from the beat-responses than the beat-responses are among each other (Grewe et al., 2003, 2007). Response similarity was calculated using the Euclidean distance between the respective neuronal responses. Firing rates were estimated using kernel-density estimates using Gaussian kernels (see methods, Equations 8–10). The standard deviation of the kernel defined the temporal resolution of the measure and could be systematically varied. We chose this distance estimation approach for its simplicity, sensitivity, and robustness. It avoids binning and temporal precision can be varied by choosing the width of the kernel. Alternative approaches using different kernels (van Rossum, 2001) or the Victor & Purpura distance (Victor and Purpura, 1997) are very likely to yield similar results (Paiva et al., 2010).

**Figure 3:**
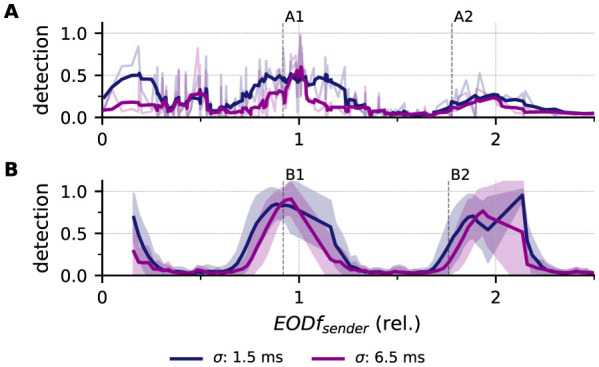
Chirp detection performance in real and model P-units is repetitive. **(A)** Detection performance as a function of the sender's *EODf* expressed relative to *EODf*_*receiver*_ to allow across-animal averaging. The detection performance is the so-called determinant, i.e., the area between the bisecting line and the ROC curve normalized to the range 0–1 where zero indicates that the chirp response is indistinguishable from the beat-response while a value of one indicates perfect detectability of the chirp. ROC analyses were performed on the firing rates estimated with different temporal precision (blue and purple). Thin lines are the averaged raw detections based on n = 64 cells but none of the cells contributed to all stimulus conditions. Thick lines are smoothed versions thereof. Analysis is based on data in which the chirps happened at the trough of the beat. Chirps had a size of 100 Hz and the sender's EOD induced AMs of 20% contrast. **(B)** Same for *n* = 32 model cells. Dashed vertical lines and the labels A1 through B2 refer to the stimulus conditions shown in Figure 2.

Despite the quantitative difference in average detection performance in real and model P-units (Figure 3), several features were found to be well conserved: (i) The detection performance is repetitive as is the beat tuning (Figure 1). (ii) Higher temporal resolution (smaller standard deviation of the Gaussian kernel used to estimate the firing rate) led to better chirp detection performance over a wider range of sender *EODf* s. (iii) Chirp detection was best around integer multiples and weakest half-way between these. From here on, we will focus on the model responses to cover wider parts of the stimulus space than is feasible with electrophysiological experiments. There is a variety of chirps (Zupanc, 2002) that the fish produce during social interactions. Of these, we used type-1 and type-2 chirps as the most commonly observed chirp types (for example, Zakon et al., 2002; Hupé and Lewis, 2008; Henninger et al., 2018; Oboti et al., 2025). Reported chirp sizes for these chirp types, i.e., the height of the frequency excursion, are not stereotyped, so we decided to use 50 Hz and 100 Hz as the lower- and upper limits observed among type-2 chirps plus a 200 Hz chirp of equal duration as mimic of the type-1 chirp. These representatives are similar to what has been applied in other studies (Walz et al., 2014; Metzen and Chacron, 2021; Wallach et al., 2022), and it was assumed that type-1 and type-2 chirps serve different purposes in opposite-sex and same-sex interactions, respectively (e.g., Hupé and Lewis, 2008). Chirps were modeled as Gaussian frequency modulations of the sender's *EODf* and were interspersed into the sender signal.

### 2.3 Chirp detection depends on AM frequency, chirp phase, chirp size, and readout timescale

As already seen above, chirp detection depended on many factors. The most obvious, the ratio between *sender* and *receiver EODf* , induces a repetitive structure in chirp detection independent of chirp characteristics and readout timescale (Figure 3). In detail, however, there are striking differences in chirp detection performances under different conditions that will be discussed in the following paragraphs.

#### 2.3.1 Chirp phase matters

**Figure 4:**
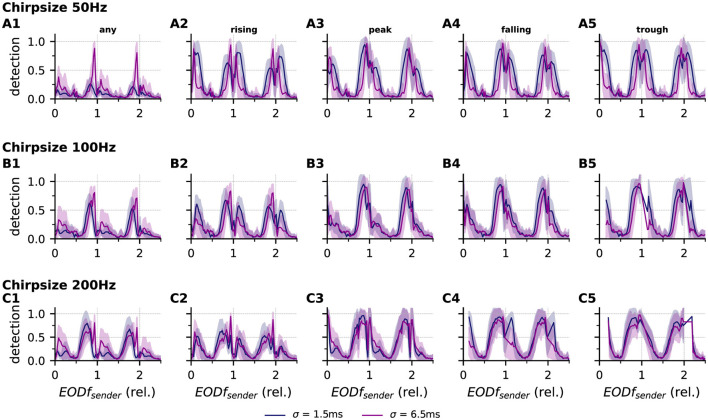
Chirp detection depends on chirp size and phase of the chirp. **(A)** Detection performances of all model cells for a 20% stimulus and a chirp size (height of the frequency excursion) of 50 Hz representative for the most commonly observed type-2 chirp with chirp sizes in the range 50 to 100 Hz. The ROC analysis was based on firing rates estimated with two different Gaussian kernels (σ of 1.5 or 6.5 ms, blue and purple curves, respectively). **(A1–A5)** represent the different chirp phases, i.e., the phase of the beat in which the chirp happened. **(B1–B5** and **C1–C5)** Same, but for different chirp sizes that represent the larger type-2 (100 Hz) and the type-1 chirp (200 Hz). Solid lines represent the average across cells, and shaded areas illustrate the standard deviation.

Chirp phase, i.e., the putative phase of the unperturbed beat in which the chirp occurs, plays a critical role. When the chirp phase was ignored (column *any* in Figure 4), the detection performance dropped to chance level for almost all conditions except for those that led to low beat frequencies, i.e., stimulus frequencies close to integer multiples of the receiver's *EODf* . At these stimulus conditions, small-chirp detection does not work on the basis of fine temporal characteristics of the responses. Rather, detection performance was high only when a rather coarse (σ = 6.5 ms) kernel was used. This is not true for the larger chirps (second and third row in Figure 4) in which the finer kernel yielded a similar detection performance as the coarser kernel. Sorting the responses according to the beat phase yielded much better detection performances. If the chirp happens on the *rising* phase of the beat, it led to a weaker detection performance across all conditions than the other phases.

#### 2.3.2 Detection performance is better for negative difference frequencies

**Figure 5:**
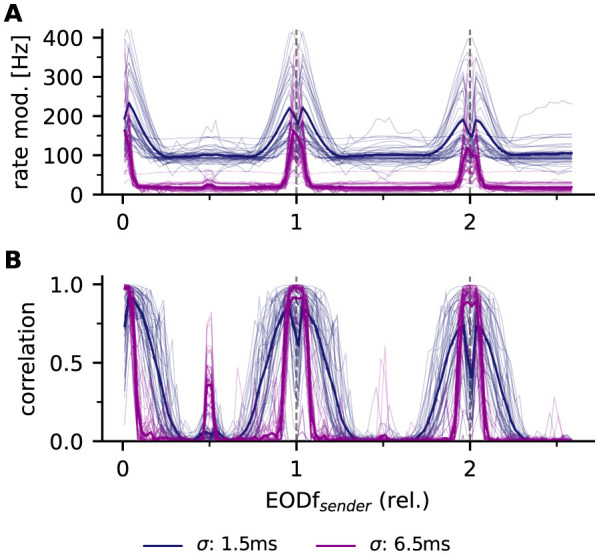
Beat tuning of the model P-units. **(A)** Tuning curve of the rate modulation as a function of the sender's *EODf* . The rate modulation quantifies how strongly the firing rate modulates around the average rate (Equation 11). It depends on the readout timescale, i.e., the width of the kernel used to estimate the firing rate. Overall, the rate modulation is stronger for finer timescales (blue lines, 1.5 ms kernel) and weaker for wider kernels (purple lines, 6.5 ms kernel) as larger kernels smear out the response. Thin lines show the tuning curves of individual models, the bold line depicts the across-cell mean. The large variability across cells reflects the strong population heterogeneity observed in P-units. **(B)** Tuning of the response correlation estimated from the pairwise correlations of repeated trials (Equation 12). Naturally, the kernel width affects this measure strongly. For larger deviations from integer multiples, the correlation drops to zero. This effect is stronger when a wider kernel has been used to calculate firing rates. Bold lines depict the mean and thin lines individual cells. Tuning curves are based on all phases.

Chirp detection performance was not symmetric around integer multiples (Figure 4). Rather, detection performances were found to be best for conditions in which the sender's *EODf* was slightly below an integer multiple. According to the chirp-encoding model proposed by Walz et al. (2014), this left shift can be expected: The beat tuning itself is symmetric around the integer multiples (Figure 5); the effect of the sender's chirp, however, is always a rightward shift as the *EODf* can only increase during a chirp. Chirps that happen in situations in which the beat is on the falling (right) flanks of the tuning curve will push the chirp response toward the valley of weak responses. On the other hand, the rightward shift imposed by the chirp may be also helpful with respect to chirp detection. If the beat happens to be in the valley between the integer multiples, a chirp-induced rightward shift can also push the AM into the tuning which then leads to elevated responses and an improved chirp detection performance.

#### 2.3.3 Timescale of the readout affects detectability

Proceeding from the model of chirp-induced transient shifts along the tuning curve, we can try to understand the observed effects. Following Walz et al. (2014), we calculated tuning curves for two response features: rate modulation (Figure 5A) and pairwise response correlation (Figure 5B, see methods for details). The resulting tuning curves are similar in two aspects: Peaks of strong tuning are centered on integer multiples of the receiver's *EODf* , and they are repetitive as expected from previous work (Barayeu et al., 2023).

Two differences stand out: (i) the peak of the correlation tuning curve is wider than the peak that of the rate modulations and (ii) there is a clear additional peak in the correlation tuning curve at a sender *EODf* of 0.5 times the receiver's *EODf* . This “intermediate” peak is most prominent when a wider kernel was used. This peak may be responsible for the slightly elevated chirp detection performance at this relative beat condition that can be seen in Figure 5A.

#### 2.3.4 Chirp size changes the shape of the detection performance

A chirp produced by the sender will always shift the response rightwards along the tuning curves. The size of a chirp defines how far this shift is. It is therefore conceivable to expect that different chirps lead to more or less salient response changes. The figures above show the detection performances as a function of the relation between the receiver's and the sender's *EODf* s. In a range from 0.5 to 1.5 times the receiver's *EODf* , this normalization makes the sender's *EODf* comparable across animals which may have different *EODf* s. If the sender's signal has a frequency that is outside this range, or if the chirp pushes it outside of this range, this is no longer true. Beyond a difference of 0.5 times the receiver's *EODf* , frequency folding will occur. The folded frequency is given by *f*_*f*_ = |*f*_2_−*f*_1_·⌊*f*2/*f*1⌉| where *f*_1_ is the receiver's *EODf* , *f*_2_ is the sender's *EODf* , and ⌊·⌉ indicates integer rounding.

**Figure 6:**
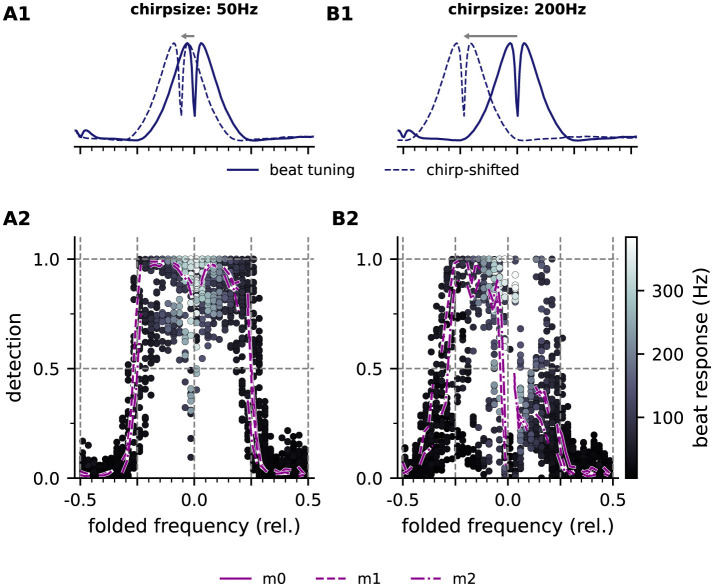
Chirp detection performance on a folded-frequencies axis of small **(A)** and big **(B)** chirps. **(A1)** schematic illustration of the chirp effect. The solid blue line shows the beat tuning around the first integer multiple. The broken line sketches the effect of the chirp, which essentially is a shift of the tuning curve to the left. The difference in response level for the beat- and the chirp tuning curves at the same folded beat frequency can be interpreted as a proxy of the chirp detectability (Walz et al., 2014). Please note that this is a sketch depicting the average tuning curve. The exact shift on the relative folded frequency axis depends on the individual *EODf* which is not considered for the purpose of this illustration. **(A2)** Small-chirp detection performance as a function of the folded beat frequency. Note: for simplification we display those folded frequencies as negative that are left of the integer multiples even though the folded frequency itself is unsigned. The color indicates the strength of the cellular response at the respective beat. Purple lines show the running median for the three sections around the integer multiples (m0: zeroth, m1: first, and m2: second multiple) that are highlighted in Figure 1B. Across these sections, median detection performances are not distinguishable, and the lines are almost perfectly on top of each other. The stimulus contrast was 20%, and the firing rate was estimated using a 1.5 ms kernel. The plot shows detection performances from all four chirp phases. **(B1, B2)** The same but for a big chirp. Note: The detection data shown in this plot are constrained to the *peak* phase.

**Figure 7:**
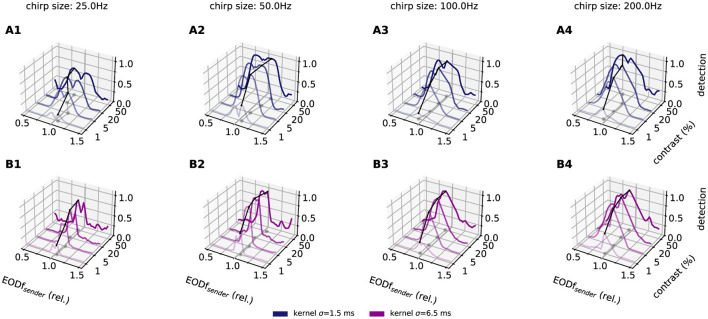
Contrast dependency of chirp detection. **(A1–A4)** Chirp detection performance for different chirp sizes and different contrasts for beat frequencies around the first integer multiple of the receiver's *EODf* . Different shading illustrates the contrast from very faint (1 %) to opaque (50 %). The chirp phases were limited to the trough of the unperturbed beat. Similar results were obtained with the other phases. The firing rates were obtained using a Gaussian kernel with a standard deviation of 1.5 ms. Black solid line connects that peak detections for the different contrasts, and the gray solid line and the dot markers on the floor of the plots mark the positions of the detection peaks. **(B1–B4)** Same as **(A)**, but for a coarser readout. The firing rates were estimated using a Gaussian kernel with a standard deviation of 6.5 ms.

Figure 6 compares chirp detectability of small, type-2, and big, type-1, chirps by re-plotting the data on a folded-frequency axis relative to the receiver's *EODf* . This allowed for comparisons across stimulus ranges that are centered on the different integer multiples. Even though the folded frequency is unsigned, we plotted those folded frequencies as negative that were left of the multiple to improve accessibility. The chirp-induced rightward shift of the sender's *EODf* led to a virtual shift to the left as sketched in Figures 6A1, B1. The detection performance has a slightly different shape than the beat tuning curves but the virtual chirp shift predicts the left shift of chirp detection well (compare panels A2 and B2 in Figure 6). The shape of the detection performance curve is more “square-like” which is due to the fact that detection performances are bounded at 1. For weaker stimuli, these curves are more similar to the beat or correlation tuning curves (Figures 5, 7).

For both chirp sizes, chirp detection is the same irrespective of the multiple which is in line with the repetitive nature of the tuning and the idea that the chirp is essentially a transient shift along this tuning curve (Walz et al., 2014). The coloring of the scattered dots in Figure 6 represents the response modulation during the beat (before the chirp happens).

For small chirps, beat responses are strong for low folded frequencies and can coincide with high detection performances. There are, however, also instances when strong beat responses only yield intermediate to low detection performances. For slightly higher folded frequencies, toward ±0.25 relative folded frequency, the chirp detection performance is still high even though beat responses are weak. On the left flank, this is caused by the chirp shifting into the tuning. On the falling flank, the chirp-induced shift moves even further out of the tuning and the fall-off is steeper than on the left flank. In the tuning valleys toward ±0.5, there is nothing to gain by a transient chirp shift, the chirp does not move the momentary amplitude modulation frequency into or out of the tuning, chirp and beat responses will both be weak, and there will be no detectable difference.

For large chirps, the pattern was more complex. The range of strong beat responses is still clustered around the zero folded frequency. Chirp detectability, however, becomes much more asymmetric. For negative folded frequencies, chirp detection is high and the folded frequency range of detectable chirps is shifted leftwards (toward −0.5) in line with the larger frequency excursion of the chirp. On the positive folded frequency side, we first see a drop of detection performance which is followed by a second peak. This second peak most likely results from the chirp-induced shift into the tuning peaks at odd multiples that are seen in some model cells (Figure 5).

#### 2.3.5 Chirp detection depends strongly on contrast

Electrocommunication suffers from what some people refer to as the “curse of the dipole field”, i.e. the EOD amplitude drops with a power of three of the distance (Benda, 2020). The amplitude drop by distance is modeled here by the different contrasts, i.e., the AM amplitude relative to the receiver's EOD amplitude. So far we focused on a contrast of 20 % which is a standard contrast widely used in experiments (for example, Benda et al., 2005, 2006; Walz et al., 2014; Metzen et al., 2016; Metzen and Chacron, 2017; Metzen et al., 2020) and is well in the range of naturally occurring contrasts when animals freely interact (Fotowat et al., 2013).

The chirp detection performance depends strongly on contrast (Figure 7). Independent of chirp size, the detection performance is already saturated at contrasts of 20%. The detection performance at contrasts as low as 1%, approximating inter-animal distances of about 30 cm (Henninger et al., 2018), drops to almost chance level. With lower contrasts, the width of the detection peak around the first integer multiple is narrower than at higher contrasts. This is in line with the tuning curves in Figure 5, the further the AM frequency is away from the integer multiple, the weaker is the response, and, accordingly, stronger stimuli are needed to drive the neuron.

##### 2.3.5.1 Chirp detection depends on the readout timescale

The readout timescale has a strong effect on the detection profile. While peak detection performances are not strongly affected even at contrasts that do not lead to a saturation of the detection performance (1% and 5%, Figure 7), detection peaks are much wider for finer versus coarser kernels. This is particularly true for smaller chirps (25 and 50 Hz frequency excursion, compare panels A1 vs. B1 and A2 vs. B2 in Figure 7).

### 2.4 Predicting chirp detection from static tuning curves

Chirps are transient increases of the sender's *EOD* frequency. In the limited range used previously, responses to chirps could be well predicted from shifts on the tuning curves (as illustrated in Figures 1, 5, Walz et al., 2014). This also extends to frequency ranges in which folding at the Nyquist frequency arises (Figure 6). To assess how tightly the detection approach pursued here is linked to changes in response rate or response correlation, we compared the predictions based on either response feature with the observed chirp detection performances that is agnostic of what changes in the responses but only signals that there are detectable chirp-induced changes.

**Figure 8:**
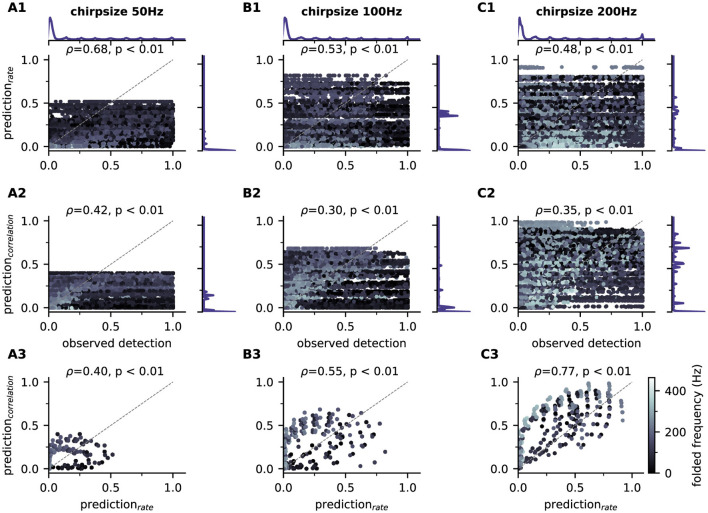
Link between detection analysis and predictions based on rate modulation and across-trial correlation. **(A1)** Predictions of chirp detectability of a 50 Hz chirp on basis of the rate modulation (see methods) plotted against the observed detection performance. The color code illustrates the folded beat frequency. Marginal plot on top shows the distribution of observed detections, and right marginal plot depicts the distribution of predicted detection. ρ and p are the Pearson correlation coefficient and the significance of the correlation. **(A2)** The same but for a prediction based on across-trial correlation. A3 Correlation of the two predictions. Y-ranges in A1, A2 are limited by chirp size: As our prediction is simply based on the difference between beat and chirp response level, the maximum achievable difference is limited by the chirp size (see Methods, Equation 13). As the tuning of the rate modulation is steeper, maximum prediction values are a little larger. Further note, clustering along the y-axis is imposed by the finite step size along the beat axis of the tuning curves and that predictions are based on the average tuning curve. **(B1–B3)** The same for a chirp size of 100 Hz. **(C1–C3)** The same for a chirp size of 200 Hz, i.e., a big chirp.

Predictions based on rate modulation (Figure 8 top row) are positively correlated with the observed chirp detection performance. These correlations drop slightly with increasing chirp size (Figures 8A1–C1). In this analysis, we used all observations originating from all cells, all beats, and all different phases. The marginal plots show, however, that most of the detections are clustered in the lower-left corner of the scatter plot (low observed and low predicted performance). The color code illustrates the folded beat frequency, and there is no obvious dependency on the folded frequency. The y-range of the predictions is bounded at different levels for different chirp sizes. This is because a chirp of a given size can only lead to a certain difference in respective response feature and thus limits the range of observable predictions (see Methods, Equation 13). We can observe that there is quite some correlation between prediction and observation (at maximum ρ = 0.68, and *R*^2^ = 0.46), that is, 46 % of the total variance can be explained from the rate modulation alone. Even though the range of predictions increases with chirp size, the correlation becomes weaker. The rate modulation alone is apparently not sufficient to explain chirp detectability.

Predictions based on response correlation yield similar results (Figures 8A2–C2). The correlation coefficients are generally lower (ρ = 0.42 at maximum, *R*^2^ = 0.17) than above and decrease with chirp size. The y-range of predictions based on response correlations is narrower as the respective tuning has a shallower profile than the tuning based on rate modulation (compare Figure 5). The coloring, i.e., the folded beat frequency, however, shows a structure. Predictions of chirp detection at high folded frequencies are larger, above the diagonal, than the observed detections, and this is true for all chirp sizes which makes intuitive sense as high folded frequencies lead to tighter spike-time locking, i.e., higher correlations.

The predictions based on the two response features are correlated with each other which is an expression of overlapping tuning curves. For larger chirp sizes, correlation increases, but predictions based on across-trial correlations predict higher detection performances than predictions based on rate modulations. This is especially true for high folded frequencies (lighter coloring). The tuning curves of the two response features overlap considerably, but the correlation tuning curve is wider. That is, detections could also be based on a combination of change in rate modulation plus a change in correlation. We tested this by fitting a standard linear regression model (see methods) that includes an interaction term. The results are shown in Table 1. For the small 50 Hz chirp, the model tells us that combining both response features is beneficial for predicting chirp detectability. The rather strongly negative interaction term indicates that combining both features is detrimental when values are high: With increasing chirp size, the coefficient of determination (*R*^2^) goes down a bit but across all chirp sizes a minimum of 50 % of the variance can be explained by a linear combination. Generally, we can thus say that a good fraction of the detection performance can be explained by the predictors based on firing rate modulation and correlation. With increasing chirp size, emphasis on rate modulation goes down and even turns negative. Detection is then best predicted based on response correlation alone. We also see that the predictors are not independently additive but their combined influence is suppressive when both predictors are high. However, the level of unexplained variance is considerable. This may be partially attributable to the signal-detection approach as detection performances are bounded and cannot exceed 100% correct. If perfect discrimination can be achieved at a tuning difference below 1.0 (the min-max difference in the normalized tuning curves), the prediction value will increase while detection performances saturate.

**Table 1:** Results of the linear regression model analysis.

chirp size	β_0_	β_1_	β_2_	β_3_	*R* ^2^
50 Hz	0.11	0.94	1.78	−4.24	0.62
100 Hz	0.16	0.18	1.99	−3.96	0.49
200 Hz	0.27	−0.23	1.66	−2.34	0.53

A substantial part but not the full detection performance can be explained by a linear model of the form $d=\beta_{0}+\beta_{1}x_{1}+\beta_{2}x_{2}+\beta_{3}\left(\left(x_{1}-\bar{x_{1}}\right)\cdot \left(x_{2}-\bar{x_{2}}\right)\right)$ that combines predictions based on rate modulation (*x*_1_) and correlation (*x*_2_) plus a mean-centered interaction term (see methods for details).

## 3 Discussion

Sensory encoding of electrocommunication signals is a multidimensional problem. Chirps come in different flavors, happen in different social contexts, and signals are heavily affected by variations in spatial distance between the interacting animals. The resulting stimulus space is too large to be sampled in electrophysiological experiments. In this study, we have applied model simulations of p-type electroreceptor afferents to support and extend electrophysiological experiments. We used a signal-detection approach in which we assume that information about chirp occurrence is available to the system when chirps induce responses that are detectably different from responses to the beat without a chirp. Previous studies focused on selected stimulus parameters, and a range of social backgrounds that was, as was later found, too narrow.

Whether and how social signals in the full behaviorally relevant range are encoded in the electrosensory periphery is addressed here. We investigate whether chirps that occur in front of high-frequency backgrounds can be encoded at all. This question became relevant as it was demonstrated that chirping at extremely high beat frequencies plays a pivotal role during courtship behaviors observed in the wild (Henninger et al., 2018). We describe that chirp encoding follows the repetitive nature of the beat encoding that we studied previously (Barayeu et al., 2023). The repetitive tuning is a consequence of frequency folding at the Nyquist frequency of the receiving fish's *EOD*, an effect that is also known as temporal aliasing in sampling theory. Chirp encoding follows this repetitive structure, and phases of good chirp detectability are observable when the sender's *EOD* frequency is in a certain range around integer multiples of the receiver's *EODf* . We find this in both experimental data and P-unit model neurons. With model simulations, we are then able to investigate chirp detection performance in dependence of chirp parameters, context, signal amplitude, and readout.

### 3.1 Frequency folding leads to individual AM contexts during courtship

During interaction, the EODs of wave-type weakly electric fish superimpose. The most striking effect is that each animal's field is amplitude-modulated through the other fish's electric field. The AM frequency depends on the relation between the individual EOD frequencies, and the P-unit responses depend on the AM frequency. Slow AMs, frequencies below 200 Hz, are well responded to, while faster AMs lead to weak or no responses. The repetitive AM tuning leads to peaks of strong responses around integer multiples of the receiver's *EODf* and valleys of weak responses in between (Figure 5).

We show that chirp responses can be predicted from chirp-induced transient shifts along this AM tuning curve. For a chirp to be detectable, it needs to move the AM from a range of strong responses toward frequencies of weak responsiveness or vice versa. Behavioral observations in Henninger et al. (2018) raised the question whether and how courting animals are at all able to sense each other given the electrosensory context in which the courting happened. We will use data from one of the observed courting dyads to illustrate the complexities of the electrosensory inputs during natural interactions and social signalling and derive hypotheses of chirp detectability under these circumstances.

Both animals sample the environment with very different EOD frequencies (Table 2) which is a consequence of a sexual dimorphism in *EODf* s. From the male's perspective male, the female's *EODf* is only 0.6 times its own frequency (*EODf*_*female*_/*EODf*_*male*_) while the male's *EODf* is 1.66 times that of the female. The difference between the *EODf* s is large (|Δ_*f*_|= 414 Hz) and far outside the classical tuning. Both frequencies are in the valley of weak encoding to the right and left of the first integer multiple of the receiver's *EODf* (compare Figure 5).

**Table 2:** Frequency folding leads to individual beat contexts.

dyad member	EODf	Nyquist frequency	rel. *EODf*_*sender*_	Δ_*f*_	Beat_*folded*_
♂	1, 035 Hz	517 Hz	0.6	−414 Hz	414 Hz
♀	621 Hz	310 Hz	1.66	414 Hz	207 Hz

EOD frequencies are taken from the exemplified courting dyad in Figure 2 in (Henninger et al. 2018). From the different EOD frequencies, it follows that beat contexts are asymmetric. Indeed, frequency folding at the individual Nyquist frequencies leads to very different folded beats. Δ_*f*_ is the prediction of the AM frequency based on the difference between the *EODf* s.

As the animals sample with different carriers and the Δ_*f*_ of 414 Hz exceeds the female's Nyquist frequency, frequency folding occurs and leads to individually different beat contexts (column Beat_*folded*_ in Table 2), actually folding the AM frequency almost back to frequencies the P-units could respond to. During the unperturbed beat, the P-units will not respond strongly and both fish will have difficulties to sense each other based on the activity of individual P-units unless animals are very close.

The effect of chirping, i.e., the transient increase in *EODf* , has asymmetric effects on the instantaneous AM frequency (Walz et al., 2014). In case of female chirps, the Δ_*f*_ will be decreased while it will be increased when the male fish chirps. As we have discussed for the beat frequencies above, we need to translate these frequencies into individual folded frequencies.

In a folded frequency regime, a 50 Hz chirp has very different effects on the individual AMs depending on the perspective, i.e., who is sender and who is receiver. If the male chirps, it will increase its *EODf* to a chirp-target-frequency of 1,085 Hz (*CTF*_*abs*_; top row in Table 3). In this example, the female is the receiver and the unperturbed folded beat frequency is 207 Hz, leading to a weak response. During the male's chirp, however, the folded chirp-target-frequency (*CTF*_*folded*_) actually goes down to 157 Hz, a frequency to which the P-units should respond well. The female should be able to detect the male chirp. Inverting the direction, i.e., the male receives the female chirp (second row in Table 3), will not lead to a detectable chirp as both frequencies, the folded beat and the folded chirp-target-frequency, are in the valley of weak responsiveness. Increasing the chirp size beyond 200 Hz, however, should lead to detectable chirp responses also in the male's P-units. Interestingly, the chirp sizes, as estimated from the spectrograms in Henninger et al. (2018), seem to not exceed 200 Hz by much. In that study, however, it was shown that males echo female chirps, while female fish do not time their chirps with respect to male chirps.

**Table 3:** Chirping announces the male to the female.

Chirp size	Sender	*CTF*_*abs*_	Receiver	Beat_*folded*_	*CTF*_*folded*_	Est. detectability
50 Hz	♂	1, 085 Hz	♀	207 Hz	157 Hz	↑
	♀	671 Hz	♂	414 Hz	364 Hz	—
100 Hz	♂	1, 135 Hz	♀	207 Hz	107 Hz	↑↑
	♀	721 Hz	♂	414 Hz	314 Hz	—
200 Hz	♂	1, 235 Hz	♀	207 Hz	7 Hz	↑↑
	♀	821 Hz	♂	414 Hz	214 Hz	↑

Effects of different chirp sizes on the estimated chirp detectability in P-unit responses of the interacting dyad in Table 2. During the chirp, the the sender's EODf approaches the Chirp-Target-Frequency (*CTF*_*abs*_). This folds at the respective Nyquist frequency of the receiver and yields a folded chirp-target-frequency (*CTF*_*folded*_). Estimated detectability is based on the changes of the response rate and across-trial correlation for the folded beat and folded chirp-target-frequency.

How the male detects the presence of the female in the first place cannot be directly inferred from the P-unit tuning. It needs to be taken into account that this may be a multisensory process involving other sensory modalities or also the second active electrosensory pathway, the timing pathway based on the *t*-type electroreceptor afferents (T-units) which provide timing information and play a critical role in controlling the jamming avoidance response by providing information about the sign of the beat (e.g., Rose and Heiligenberg, 1986). Furthermore, our predictions are based on the average P-unit tuning. The population of P-units, however, is heterogeneous (Nelson et al., 1997; Grewe et al., 2017; Hladnik and Grewe, 2023) and some P-units show elevated beat responses even in the valley between the integer multiples (thin lines in Figure 5) and may still signal the occurrence of a chirp.

The courtship interactions happen at close distances at which the strength of the signals is large (Henninger et al., 2018). As we have seen above, changes in the relative amplitude, i.e., the contrast, strongly affect the beat range over which a chirp can be detected. With large contrasts, the range of high chirp detectability is much wider than under low-contrast conditions. Strong stimuli under close-distance interactions may allow the communication partners to electrically sense their chirping.

Why the sexual dimorphism in male and female *EODf* s evolved despite that it complicates sensing under circumstances that have a direct effect on fitness, courtship and reproduction, remains speculative. Increasing stimulus intensities improved chirp detectability. Electric behavior at the limit of the sensory capabilities might thus ensure that the courting partners are actually in close proximity which will increase the likelihood of successful external fertilization. More research is required to approach such questions. Models of the electrosensory periphery will prove essential in this endeavor.

Classically, behavioral responses to foreign signals have been characterized only for stimuli that were close the recorded animal's *EODf* . A few other studies, however, reported that *A. leptorhynchus* shows chirp responses to stimuli of very low frequency (Dunlap et al., 2010) as well as to the electric organ discharges of heterospecifics even if their *EODfs* were far away from the receiving animal's *EODf* (Dunlap et al., 2010; Fugère and Krahe, 2010). In particular in the study by (Dunlap et al. 2010), stimuli were outside the range of highest sensitivity (Hopkins, 1976) and difference frequencies between the sender and receiver exceeded 250 Hz. Accordingly, the receiving animal should not be able to perceive these signals and should not respond. The power spectra of EOD waveforms of some electric fishes show a distinct harmonic structure (e.g., Stoddard and Markham, 2008; Benda et al., 2013) and across-species responses might be working when one the harmonics falls into the tuning of the receiving animal. It might also be that frequency folding as described here leads to sensory responsiveness and thus to the observed behavioral responses. This is in line with the jamming avoidance responses described in Barayeu et al. (2023) which also occurs when the stimulus frequency is close to the zeroth integer multiple of the receiver's *EODf* . Behavioral response in this range were shown to be weaker than at higher multiples, but this might be compensated for by an increased stimulus amplitude.

Above we stated that large frequency difference were largely ignored in studies on beat and chirp encoding in P-units until our own observations among freely behaving animals. While formally correct, Savard et al. (2011) report responses of P-units to high-frequency band-limited noise stimuli with spectral power in the range 375 to 425 Hz. In contrast to *direct* stimulation with mimics of conspecific signals (*EOD*_*receiver*_+*c*·*EOD*_*sender*_, with *c* being the strength of the foreign signal, i.e., the contrast), amplitude-modulation stimuli are created by adding an amplitude modulated version of the self-generated field {*EOD*_*receiver*_·[1+*am*(*t*)], where *am*(*t*) is the desired amplitude modulation}. The stimulation is thus somewhat different, and the spectral contents of the combined signal depend strongly on the *EODf* of the receiver (see also Supplementary Figure 2). What actually drives the neuron is a rectified version of the combined signal and most likely contains spectral power in lower-frequency ranges that drive the P-units. From inspection of Figure 4 and the available information on the *EODf* s of the recorded animals and the relatively strong stimuli (30% contrast) in Savard et al. (2011), is seems plausible that there are always lower-frequency spectral components evoking responses. How the effects shown in Supplementary Figure 2 affect the cross-spectrum, and thus, the stimulus-response coherence is beyond the scope of the present study but needs to be addressed in future studies.

### 3.2 Relevance of chirp phase

Chirp responses in P-units were found to depend on the timing within the beat cycle (Benda et al., 2005; Walz et al., 2014; Metzen and Chacron, 2017, see also Supplementary Figure 1). This effect is most pronounced at slow beat frequencies, where the same chirp can elicit opposite changes in firing rate depending on whether it occurs at the peak or trough of the beat. The beat, however, is not the same across the body surface. At a given instance in time, the headward dipole lobe is positive relative to its counterpart (Assad et al., 1998) and superposition with the sender's EOD will lead to beats that are phase-shifted about 180 degrees. Accordingly, the chirp will be in opposing phases of the beat. Furthermore, the spatial arrangement of interacting animals affects how different body parts are exposed to the foreign EOD. It will have opposing effects on ipsi- and contralateral sides and again, the beats on both sides are phase-shifted by 180 degrees.

Postsynaptic neurons on the next level of processing in the electrosensory lateral line lobe (ELL, see below) integrate P-unit input and have receptive fields of varying sizes (Maler, 2009). A neuron that integrates information originating from head and tail regions would likely show a weak chirp detection performance, while a neuron with a localized receptive field could show a good chirp-detection performance. In controlling the jamming avoidance response, comparing phase modulations at different body locations is key for estimating the direction of the behavioral response (Heiligenberg, 1989). It seems plausible that the same across-body comparisons might also play a role in chirp detection. There are commissural connections in the ELL (Bastian et al., 1993) that could mediate this. Especially, it was concluded that ovoid cells participate in a common mode rejection mechanism that would emphasize differences between the activity in both hemispheres (Bastian et al., 1993), but their role in chirp detection has not been investigated yet and might strongly depend on the beat frequency.

### 3.3 Chirps as probing signals

Until recently, it was clear to the community that chirps are communication signals. Early studies, for example by Hagedorn and Heiligenberg (1985), describe them in the context of aggression and courtship. In *A. leptorhynchus*, they can be evoked by stimulating the animal with a mimic of another fish's *EOD* in so-called chirp-chamber experiments (e.g., Zupanc and Maler, 1993). Later, type-2 chirps were concluded to be an appeasement signal that the submissive animal emits to avoid being attacked (Hupé and Lewis, 2008) while other studies found more chirping in dominant fish and concluded them to be signal of dominance and aggression (Triefenbach and Zakon, 2008). Recordings of courtship and aggressive encounters in freely behaving animals by Henninger et al. (2018) confirmed these roles of chirping.

The beating AMs that arise from the superposition of the EODs of interacting animals are an ambiguous signal. The frequency of the AM, ignoring frequency folding for the moment, is the absolute difference between the individual *EODf* s. At the same time, the sign, i.e., whether the receiving fish's EOD frequency is above or below that of the other animal, is an important cue as it contains information about the hierarchical status (e.g., Raab et al., 2021). Walz et al. (2014) suggested that the animals could use a small chirp to estimate the sign of the beat, i.e., whether the other animal has a higher or lower *EODf* , as the chirp response will be different depending on the relation between receiver and sender *EODf* .

Recent work by Oboti et al. (2025) casts further doubts on the role of chirps as communication signals. In their work, a vast number of chirps have been recorded in a variety of behavioral settings. The authors could not find significant correlations between chirping activity and behavior. They further observed that significantly more chirps are emitted in cluttered environments when the assessment of the putative communication partner is difficult. They concluded that the chirp does not serve to transfer information to the receiver but as an active sensing method employed by the sender (Oboti et al., 2025). Subsequent work provides further evidence in this direction by describing increased information in the electric images and an improved foreign-fish detection based on electrosensory responses while chirping (Oboti et al., 2026, unpublished observations).

Spike-frequency adaptation in the electrosensory periphery acts as a high-pass filter (Benda and Herz, 2003) that reduces the responses to low-frequency beats (Benda et al., 2005). The transient frequency excursion of the chirp disrupts the continuous AM pattern and makes the chirp response stand out (Benda et al., 2005, 2006; Walz et al., 2014). On the first level of central processing in the hindbrain, feedback projections reduce the responses to low-frequency beats (Bastian et al., 2004) and a disruption of the ongoing beat increases the saliency of the chirp (e.g., Marsat and Maler, 2012, see also below). The chirp could thus be actively used to transiently move the sensory input out of the adapted state and to listen to telltale changes in the electrosensory input that are indicative of the presence of another animal.

On the other hand, chirps are only very rarely observed when animals are alone and not electrically stimulated. Second, the amount of chirps that are produced depends on the AM frequency and the sex of the animal (Zupanc and Maler, 1993; Hupé and Lewis, 2008). Female fish chirp much less than male fish, but why would they have less need for disambiguation of the electrosensory context? At the same time, our results show that especially the larger chirps would be a way to escape the valleys of weak encoding between the optima of the tuning and to transiently shift the AM into a range that is encoded in one or the other response feature. If the animals were using larger chirps to escape these valleys, the chirp size should depend on the beat situation. So far, there is no experimental evidence for this (see also Supplementary Figure 1 in Oboti et al., 2025).

### 3.4 Encoding of chirps in higher brain areas

Chirp detectability strongly depends on the contrast, i.e., the relative amplitudes of sender and receiver. When the sender's signal is weak, the sender's chirps are hard to detect in the responses of single P-units. This relates to the detection performance and also to the range of beat frequencies for which detections are possible (Figure 7). In our simulations, we only went down to 1% contrasts which relates to inter-animal distances of 30 cm at maximum, which relates to roughly 1.5 times the fish's body length (Fotowat et al., 2013; Yu et al., 2019; Henninger et al., 2018; Benda, 2020).

The electric images resulting from EOD superposition are global. That is, large parts of the body, and thus the sensory surface, are stimulated simultaneously. Hence, the neuronal activity of large populations of electroreceptors contains similar information. Our analyses here are based on single cells, but a population approach could be used to increase sensitivity and increase the range of spatial distances over which communication signals could be perceived. P-type electroreceptor afferents carry electrosensory information to the brain. They project into the medullary electrosensory lateral line lobe (ELL, e.g., Krahe and Maler, 2014) where they trifurcate and provide feed-forward input to pyramidal cells in three adjacent maps. The pyramidal cells come in different flavors (Marsat and Maler, 2010), have different tuning properties (Chacron et al., 2005; Krahe et al., 2008), and receptive field sizes (Maler, 2009). On the extremes, pyramidal cells in the centro-medial segment integrate tens of P-units while those in the lateral segment integrate input from up to 1,000 P-units (Maler, 2009). While the sensitivity may be increased by convergence, integrating over larger receptive fields will also smear out the fine temporal details of the afferent responses due to spread of conduction delays (Hladnik and Grewe, 2023) and could thus limit the beat range in which chirps can be detected as our results predict that coarser kernels used for readout lead to narrower tuning curves. Previous studies on chirp coding in the ELL investigated the role of different cell types and burst firing (e.g., Marsat and Maler, 2010, 2012) or followed the encoding of small chirps along the hierarchy from afferents via hind- and midbrain neuron to explain the invariant behavioral echo responses to foreign chirps (Metzen et al., 2016; Metzen and Chacron, 2017; Metzen et al., 2020). These studies had a different goal and accordingly focused on rather narrow ranges of social contexts and employed relatively strong stimuli. It is thus an open question if convergence in the ELL would support chirp detection in regimes of weak stimuli.

In the torus semicircularis in the midbrain, sub-populations of neurons respond very much like their presynaptic inputs while other cells respond in a sparse and robust manner to specific chirp types (Vonderschen and Chacron, 2011; Metzen and Chacron, 2021). In the sense of chirps being probing signals and chirp sizes being intentionally varied to escape the regimes of weak beat tuning, representing big and small chirps with distinct neuronal populations does not seem meaningful. In the thalamic preglomerular nucleus in the forebrain, it is hypothesized that a multidimensional space is established in which the dimensions stand for different aspects of the electrosensory environment (beat frequency, self- vs. foreign chirp, motion direction, i.e., approaching or receding, and possibly others). This space may then be used to interpret afferent and top-down information in a combinatorial way to evaluate the electrosensory scene and control the proper behaviors (Wallach et al., 2022). A combinatorial representation that includes the beat frequency, i.e., the context of the chirp, suggests an importance of this piece of information which, to some extent, contradicts the idea of chirps merely having a pure probing function to detect the presence of a foreign fish. On the other hand, probing and communication functions are not mutually exclusive.

### 3.5 Conclusion and future prospects

Using a combined approach of electrophysiology and modelling allows us to study electrosensory information processing and the encoding of chirps at a level of detail that is hardly possible with experimental approaches alone. Experimental data show that chirp detection shows the same repetitive structure as the beat tuning. Chirp responses in real and modeled P-units are very similar, and the model also reproduces the repetitive chirp encoding. The basis for the repetitive encoding is the frequency folding that enables the P-units to respond to high-frequency stimuli that are far outside the classical tuning. At the same time, frequency folding leads to surprising effects in the spectral content of the electrosensory inputs as we exemplified at the example data from freely behaving animals. The male's chirp, even though it transiently increases the frequency difference between male and female *EODf* s, can lead to reduced AM frequencies which then leads to a good detectability of the chirp. Actually, the folded beat frequency and, maybe even more importantly, the folded chirp-target-frequency depend on the frequency of each fish's *EOD*, is thus highly individual.

Still, with this study we barely scratch the surface. The combination of modeling and electrophysiology is the test bed for deeper analyses. Future studies should include population coding and the effects of convergence and heterogeneity. Furthermore, different readout mechanisms based, for example, on bursting or synchronous activity could be considered (Sharafi et al., 2013; Schlungbaum et al., 2025). Different species of electric fishes, even though closely related, employ different chirp types. Similarly, the encoding of different chirp (sub-)types could be considered in a comparative approach. The present study must be seen as a first but necessary step toward a full understanding of chirp encoding in the electrosensory periphery.

## 4 Methods

Experiments were carried out on 13 specimens of *Apteronotus leptorhynchus* of either sex. Experimental procedures complied with European and national law and were approved by the Ethics Committee of the local government (Regierungspräsidium Tübingen, permit no ZP 1/16). Animals were obtained from a commercial supplier for tropical fish (Aquarium Glaser, Rodgau, Germany). Animals were kept in groups of varying sizes at water temperatures of 25 ±1°C and a water conductivity of approximately 270μ*S*/*cm* under a 12 -h:12 -h light–dark cycle.

### 4.1 Experimental procedures

P-units were recorded in the posterior branch of the anterior lateral line nerve. The nerve was exposed by making a small cut into the skin just dorsal of the operculum where the nerve runs directly beneath the skin before descending to the anterior lateral line nerve ganglion. Prior to surgery, the animals were deeply anesthetized by bath application of MS-222 (125 mg/L buffered to pH 7.0 with sodium bicarbonate). During surgery, anesthesia was maintained by 100 mg/L MS 222 in the respiration water. Animals were head-fixed to a glue-post attached to the exposed skull bone. The surgically affected parts of the skin were topically treated with 2%° lidocaine ointment (bela-pharm, Vechta, Germany). After surgery, fish were immobilized by injection of 50-75μ*l* tubocurarine (1 mg/ml, Sigma-Aldrich) dissolved in fish saline. Fish were then transferred into the experimental tank, and respiration was switched to normal tank water. Animals were given enough time to recover from anesthesia and were closely monitored for indications of stress or insufficient oxygenation throughout the whole experiment. Analgesia was refreshed every 2 h. Experiments lasted 8 h at maximum. Euthanasia was done by overdosing the anesthetic and subsequent cutting of the spinal cord.

### 4.2 Experimental setup

For the recordings, fish were positioned centrally in the experimental tank, with the major parts of their body submerged. Body parts that were not below water surface were covered with paper tissue to prevent drying of the skin. The fish's self-generated electric field was recorded with two pairs of electrodes. The first was a head-to-tail measurement of the EOD that was amplified with factors between 100 and 500 depending on the respective fish's EOD amplitude and band-pass filtered (3 to 1,500 Hz pass-band, DPA2-FX; npi electronics, Tamm, Germany). These electrodes were placed iso-potential to the stimulus to record the EOD without stimulus contamination. The second pair of electrodes was placed next to the animal just caudal of the operculum and oriented orthogonal to the fish's head-to-tail body axis. In this configuration, the recording electrodes pick up the fish's self-generated electric field and the stimulus-induced modulations of it. This signal served as a proxy of the so-called transdermal potential that the electroreceptors in the skin are exposed to. Signals were amplified and band-pass filtered (3 to 1,500 Hz pass-band, DPA2-FX; npi electronics, Tamm, Germany). Electrical stimuli were delivered into the tank via two carbon rods (30 cm length, 8 cm diameter) that were placed parallel to the animal's longitudinal body axis on both sides of the animal (15 cm distance from the animal). Stimuli were computer generated, attenuated to yield the desired intensities, and isolated from ground (ATN and ISO-STIM modules, npi electronics, Tamm, Germany). Stimuli were calibrated with respect to the self-generated electric field amplitude.

Sharp glass electrodes (borosilicate; 1.5 mm outer diameter; GB150F-8P; Science Products, Hofheim, Germany) were pulled to a resistance of 50–100 MΩ (P-97 puller; Sutter Instrument, Novato, CA) and filled with 1 M KCl solution. Electrodes were fixed in a microdrive (Luigs-Neumann, Ratingen, Germany) and advanced into the nerve. Recordings of electroreceptor afferents were 10-fold amplified and low-pass filtered at 10 kHz (SEC-05, npi-electronics, Tamm, Germany, operated in bridge mode).

All signals, neuronal recordings, recorded EOD and the generated stimulus, were digitized with a sampling rate of 20 kHz (PCI-6229, National Instruments, Austin, TX). RELACS (https://github.com/relacs) running on a Linux computer was used for online spike and EOD detection, stimulus generation, and calibration. Recorded data were then stored for offline analysis using the nix file format (Stoewer et al., 2014).

P-type electroreceptor afferents (P-units) were identified based on their characteristic response properties; baseline firing rates in the range 50–450 Hz, a clear phase-locking to the EOD, a high coefficient of variation of the baseline activity, and their responses to amplitude modulations of the self-generated EOD (Grewe et al., 2017; Hladnik and Grewe, 2023).

### 4.3 Chirp stimulation

The recorded animals were stimulated with mimics of conspecific signals that contained artificial chirps. These mimics were pure cosine waves, and frequencies were defined relative to the recorded fish's EOD frequency *EODF*_*sender*_ in the range 0.1 to 2.5 times the receiver's *EODf* . Stimulus amplitudes were selected to yield an amplitude modulation depth of 20% of the unperturbed EOD amplitude. Chirps had a duration of 14 ms, a frequency excursion of 100 Hz, and an amplitude reduction of 2 %. Frequency excursion and amplitude reduction followed a Gaussian profile.

### 4.4 Leaky integrate-and-fire model

A leaky integrate-and-fire (LIF) model was constructed to reproduce the specific firing properties of P-units (see also Chacron et al., 2001; Sinz et al., 2020; Barayeu et al., 2023). Under baseline conditions, i.e., in the absence of an external stimulus, the model was driven by the fish's own EOD modeled as a cosine wave


$$\begin{array}{l} y\left(t\right)=y_{EOD}\left(t\right)=\cos\left(2\pi f_{EOD}t\right) \end{array}$$ (1)

with the EOD frequency *f*_*EOD*_ and an amplitude normalized to ±one.

In the model, the input *y*(*t*) was first passed through a threshold to resemble synaptic transmission between primary receptor cells and afferent.


$$\begin{array}{l} {\left\lfloor y\left(t\right)\right\rfloor }_{0}^{3}=\{\begin{array}{ccc} y{\left(t\right)}^{3} & ; & y(t)\geq 0\\ 0 & ; & y(t)<0 \end{array} \end{array}$$ (2)

${\left\lfloor y\left(t\right)\right\rfloor }_{0}^{3}$ denotes a threshold operation that sets negative values to zero and the power of three mimics a further synaptic non-linearity which is required to explain the experimentally observed repetitive beat tuning (Barayeu et al., 2023).

The resulting receptor signal was then low-pass filtered to approximate passive signal conduction in the afferent's dendrite


$$\begin{array}{l} \tau_{d}\frac{dV_{d}}{dt}=-V_{d}+{\left\lfloor y\left(t\right)\right\rfloor }_{0}^{3} \end{array}$$ (3)

with τ_*d*_ being the dendritic time constant. Because the input was dimensionless, the dendritic voltage was dimensionless, too. The combination of threshold and low-pass filtering extracts the amplitude modulation of the input *y*(*t*).

The dendritic voltage *V*_*d*_(*t*) is then passed into the leaky integrate-and-fire (LIF) spike-generator


$$\begin{array}{l} \tau_{m}\frac{dV_{m}}{dt}=-V_{m}+\mu +\alpha V_{d}-A+\sqrt{2D}\xi \left(t\right) \end{array}$$ (4)

where τ_*m*_ represents the membrane time-constant, μ a fixed bias current, α a scaling factor for *V*_*d*_, *A* an inhibiting adaptation current, and $\sqrt{2D}\xi \left(t\right)$ an internal white noise with strength *D*. All state variables except τ_*m*_ were dimensionless.

The adaptation current *A* developed according to the differential equation


$$\begin{array}{l} \tau_{A}\frac{dA}{dt}=-A \end{array}$$ (5)

with an adaptation time constant τ_*A*_.

Whenever the membrane voltage *V*_*m*_(*t*) crossed the spiking threshold θ = 1, a spike was generated, *V*_*m*_(*t*) was reset to 0, the adaptation current was incremented by Δ*A*, and integration of *V*_*m*_(*t*) was paused for the duration of a refractory period *t*_*ref*_.


$$\begin{array}{l} V_{m}(t)\geq \theta \;:\{\begin{array}{ccc} V_{m} & \mapsto & 0\\ A & \mapsto & A+\Delta A/\tau_{A} \end{array} \end{array}$$ (6)

### 4.5 Numerical implementation

The model's ODEs were integrated by the Euler forward method with a time step of Δ*t* = 0.05 ms. The intrinsic noise ξ(*t*) (Equation 4) was added by drawing a random number from a normal distribution N(0,1) with zero mean and standard deviation of one in each time step *i*. This number was multiplied with $\sqrt{2D}$ and divided by $\sqrt{\Delta t}$:


$$\begin{array}{l} V_{m_{i+1}}=V_{m_{i}}+\left(-V_{m_{i}}+\mu +\alpha V_{d_{i}}-A_{i}+\sqrt{\frac{2D}{\Delta t}}N{\left(0,1\right)}_{i}\right)\frac{\Delta t}{\tau_{m}} \end{array}$$ (7)

### 4.6 Model parameters

The eight free parameters of the P-unit model α, τ_*m*_, μ, *D*, τ_*A*_, Δ_*A*_, τ_*d*_, and *t*_*ref*_, were fitted to both the baseline activity (baseline firing rate, CV of ISIs, serial correlation of ISIs at lag one, and vector strength of spike coupling to EOD) and the responses to step-like increases and decreases in EOD amplitude (onset-state and steady-state responses). The model and model parameters are available under https://github.com/bendalab/punitmodel.

### 4.7 Stimuli for the model

The model neurons were driven with similar stimuli as the experimentally recorded neurons. To mimic the interaction with a foreign animal, the receiving fish's EOD (Equation 1) was normalized to an amplitude of one and the sender's EOD was scaled to yield a given AM depth (contrast) and added to the receiver's EOD. Artificial chirps were inserted into the sender's EOD. Frequency excursion (chirp size) and amplitude drop during chirping followed a Gaussian profile.

Each simulation run began from randomly chosen start values for membrane voltage and adaptation current. Each run consisted of an 10 s initial phase in which a random noise stimulus was faded in and out. This part of the simulation was discarded. In the next 10 s, the model cell's response to the self-generated EOD was simulated. In the following 80 s, the sender's EOD was switched on.

### 4.8 Data analysis

Data analyses were performed with custom-written routines in Python 3 using the packages matplotlib (Hunter, 2007), nixio (Stoewer et al., 2014), numpy (Walt et al., 2011), pandas (McKinney et al., 2010), scipy (Virtanen et al., 2020), sklearn (Pedregosa et al., 2011), and statsmodels (Seabold and Perktold, 2010).

#### 4.8.1 Data processing

The firing rate as a function of time, *r*_*k*_(*t*), was estimated by kernel convolution of the spike responses $x_{k}\left(t\right)=\underset{i}{\sum }\delta \left(t-t_{k,i}\right)$ of trial *k* with spikes at times *t*_*k, i*_ with a Gaussian kernel:


$$\begin{array}{l} F\left(t\right)=\frac{1}{\sqrt{2\pi \sigma_{gauss}^{2}}}e^{-\frac{t^{2}}{2\sigma_{gauss}^{2}}} \end{array}$$ (8)

with σ_*gauss*_ the standard deviation of the kernel. The single trial firing is then given by


$$\begin{array}{l} r_{k}\left(t\right)=x_{k}\left(t\right)*F\left(t\right)=\overset{+\infty }{\underset{-\infty }{\int }}x_{k}\left(t^{\prime }\right)F\left(t-t^{\prime }\right)\text{d}t^{\prime }, \end{array}$$ (9)

where * denotes convolution. Averaging across *k* trials yields the firing rate (*r*(*t*)):


$$\begin{array}{l} r\left(t\right)={\langle r_{k}\left(t\right)\rangle }_{k}. \end{array}$$ (10)

In response to dynamic stimuli, the firing rate is modulated around an average firing rate. We quantified this firing rate modulation as the standard deviation of the across-trial firing rate


$$\begin{array}{l} \sigma_{mod}=\sqrt{{\langle {\left(r\left(t\right)-{\langle r\left(t\right)\rangle }_{t}\right)}^{2}\rangle }_{t}}, \end{array}$$ (11)

where 〈·〉_*t*_ denotes averaging over time, and 〈*r*(*t*)〉_*t*_ is the time-averaged firing rate.

The rate modulation was used to quantify the stimulus effectiveness in driving the neuron and served as a proxy for the neuronal sensitivity for a given stimulus.

The correlation between pairs of responses *r*_*i*_(*t*) and *r*_*j*_(*t*) (short *r*_*i*_ and *r*_*j*_) was used to quantify response synchrony:


$$\begin{array}{l} \rho_{i,j}=\frac{{\langle \left(r_{i}-{\langle r_{i}\rangle }_{t}\right)\cdot \left(r_{j}-{\langle r_{j}\rangle }_{t}\right)\rangle }_{t}}{\sqrt{{\langle {\left(r_{i}-{\langle r_{i}\rangle }_{t}\right)}^{2}\rangle }_{t}}\cdot \sqrt{{\langle {\left(r_{j}-{\langle r_{j}\rangle }_{t}\right)}^{2}\rangle }_{t}}} \end{array}$$ (12)

### 4.9 ROC analysis

We performed a ROC analysis to assess how well the chirps are encoded in the responses. For this, we extracted sets of chirp- and beat response segments from the firing rate estimates of experimental and model responses. The segment length was set to 20 ms centered on the chirp to yield the chirp segments or was placed in the unperturbed beat in the same phase as the chirps. This resulted in the same number of response segments that depended on the number of chirps that were included in the sender's EOD. The null distribution was established from the beat responses by estimating the Euclidean pairwise distance between all beat responses. The test distribution contains the Euclidean distances between each chirp response and all beat responses (see Supplementary Figure 1).

From the ROC analysis, we extract the area-under-the curve and convert it to the determinant, which is the integral between the intersection line and the ROC curve. This measure varies between 0 (indistinguishable) and 1 (perfect detection).

### 4.10 Predicting chirp detection from the tuning curves

In (Walz et al. 2014), it was shown that the responses to chirps can be well predicted by the transient shifts along the static tuning of the cells (Figure 1B). Two types of tuning curves were estimated: (i) the rate modulation, i.e., how strongly the neuronal response was modulated by the stimulus (Equation 11), and (ii) the pairwise correlations of the responses which characterizes the spike time synchrony (Equation 12).

Here, we want to test whether the changes in any of the response features (rate of correlation) suffices to predict the chirp detection performance estimated from the ROC analysis described above. The across cell averaged tuning curves were first normalized to span the range [0 .. 1]. From the average tuning curves, the response feature at the unperturbed relative beat frequency and at the frequency at the peak of the chirp were read which gives the *rate*_*beat*_ and *rate*_*chirp*_ or *correlation*_*beat*_ and *correlation*_*chirp*_, respectively. The difference between the two respective values was then interpreted as a prediction of the chirp detectability, for example:


$$\begin{array}{l} prediction_{rate}=|rate_{chirp}-rate_{beat}| \end{array}$$ (13)

This implies that the chirp detection is easiest when the difference between the response features during beat and chirp was large.

### 4.11 Linear regression model

The predictive power of the detection procedure for ROC performance was assessed using a linear regression model (*statsmodels* package). ROC chirp-detection performance (*y*) served as the dependent variable, while predictions derived from the rate and correlation tuning curves were used as predictors (*x*_1_ and *x*_2_).

To account for potential non-additive effects, we included an interaction term between the predictors. Specifically, we fitted the model


$$\begin{array}{l} y=\beta_{0}+\beta_{1}x_{1}+\beta_{2}x_{2}+\beta_{3}\left(\left(x_{1}-{\bar{x}}_{1}\right)\cdot \left(x_{2}-{\bar{x}}_{2}\right)\right), \end{array}$$ (14)

where the interaction term was computed using mean-centered predictors.

## Data Availability

The raw data supporting the conclusions of this article will be made available by the authors, without undue reservation.
