# Supplementary material for: Toward model-guided electrophysiology—Encoding of chirps in the electrosensory periphery of Apteronotus leptorhynchus
Source: Front Comput Neurosci. 2026 May 29;20:1827196. doi: 10.3389/fncom.2026.1827196 (PMC13260415; doi:10.3389/fncom.2026.1827196)
Supplement: Supplementary file 1 [file Supplementary_file_1.pdf]

Toward model-guided electrophysiology — encoding of chirps in the electrosensory periphery of *Apteronotus leptorhynchus*

Alexandra Barayeu [1], **Jan Benda** [1,2] **and Jan Grewe** [1,*]

[1] *Neuroethology, Institute for Neurobiology, University of Tübingen, Tübingen, Germany*
[2] *Bernstein Center for Computational Neuroscience Tübingen, University of Tübingen, Tübingen, Germany*

Correspondence*:
Jan Grewe
jan.grewe@uni-tuebingen.de

1 SUPPORTING INFORMATION

1.1 Discrimination method

1.2 Direct versus AM stimulation

There are two ways in which electroreceptors are typically stimulated. The first and conceptually easiest is the so-called *direct* stimulation (figure 2 A_{1-4}) in which a second signal is generated and led into the recording tank where it superimposes with the fish's self-generated field. The receiving animal will be exposed to a combined signal that shows an amplitude modulation and phase modulation (not visible here). The latter is key for controlling the jamming-avoidance response (Heiligenberg, 1989) but mostly unwanted when studying P-units that code for amplitude modulations. Spectral decomposition of the combined signal $(EOD_1 + EOD_2)$ shows two peaks at the respective frequencies. When the signal undergoes rectification (e.g. at the synapse from primary receptor to the afferent), harmonics are induced and a spectral peak at the AM frequency appears (A_4).

To evoke pure amplitude modulations without the accompanying phase modulation, one can combine the receiving fish's EOD with an amplitude modulated version of its self-generated field. This is known as the so-called *AM* stimulation $(EOD_1 \cdot (1 + am(t))$, illustrated in fig 2 B_{1-4}). The power spectrum of the resulting combined signal shows a peak at the EOD frequency which is flanked by side-peaks at *EODf* ±50 Hz. The AM frequency itself is not part of the spectrum. Rectification again induces the AM peak at the expected frequency (50 Hz, B_4).

AM stimulation allows to easily apply any desired waveform to the EOD and study the neuronal responses for example to noise stimuli which is desirable for information theoretic analyses. Figure 2 C_{1-4} shows what happens when a narrow-band noise stimulus (C_2) is combined with an EOD carrier. The combined signal itself contains a spectral peak at the EOD frequency that is flanked by the narrow-band spectra at a distance of 400 Hz (C_3). Rectification leads to the desired spectral components centered around 400 Hz. For the purpose of this illustration we chose a carrier of 600 Hz. At other carrier frequencies, the picture will look differently as the side-bands move close together at 700 Hz, or even superimpose at 800 Hz carrier

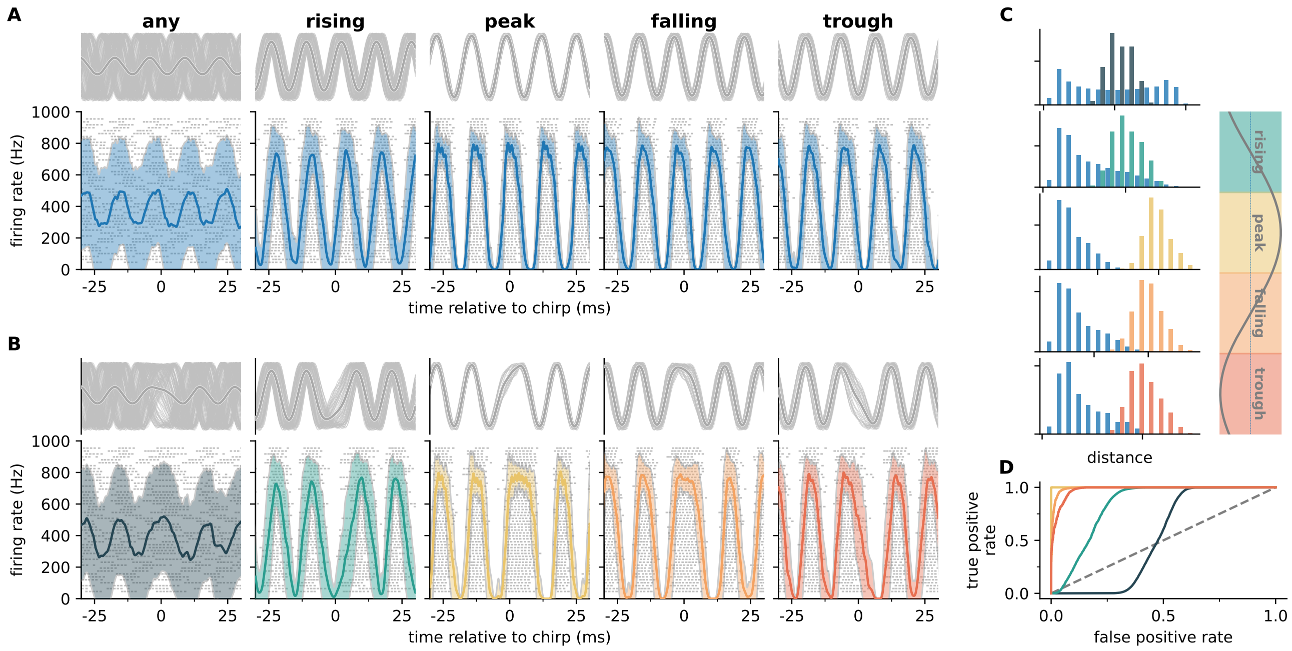

Figure 1. Response sorting according to the chirp phase and chirp detection. A AMs (top) and model responses (bottom) during the unperturbed beat, i.e. without a chirp. AM segments are centered around the "virtual" times of the chirps, i.e. at least four beat periods before the chirp happened in B. Different columns show the AMs ignoring the "virtual" chirp phase or sorted according to the phase. Solid lines are the means and shaded areas represent the standard deviation. **B** AMs and response segments centered around the chirps. **C** From the beat responses in A, we establish the null-distributions (blue) and from the responses in B the test-distributions of distances (colored distributions, see methods). The histograms show these distributions for the different analyses ignoring the chirp phase (top histograms) and sorting according to the chirp phase. The colored segment and the sine-wave illustrate chirp sorting. **D** Receiver-operating-characteristics (ROC curves) for the different analyses. Data from model cell 2011-10-25-ad-invivo-1.

frequency. The rectified signal that actually drives the P-units in these cases contains spectral power not only in the desired frequency band but also at lower frequencies.

REFERENCES

Heiligenberg, W. (1989). Coding and processing of electrosensory information in gymnotiform fish. *Journal of Experimental Biology* 146, 255–275. doi:10.1242/jeb.146.1.255

Savard, M., Krahe, R., and Chacron, M. (2011). Neural heterogeneities influence envelope and temporal coding at the sensory periphery. *Neuroscience* 172, 270–284

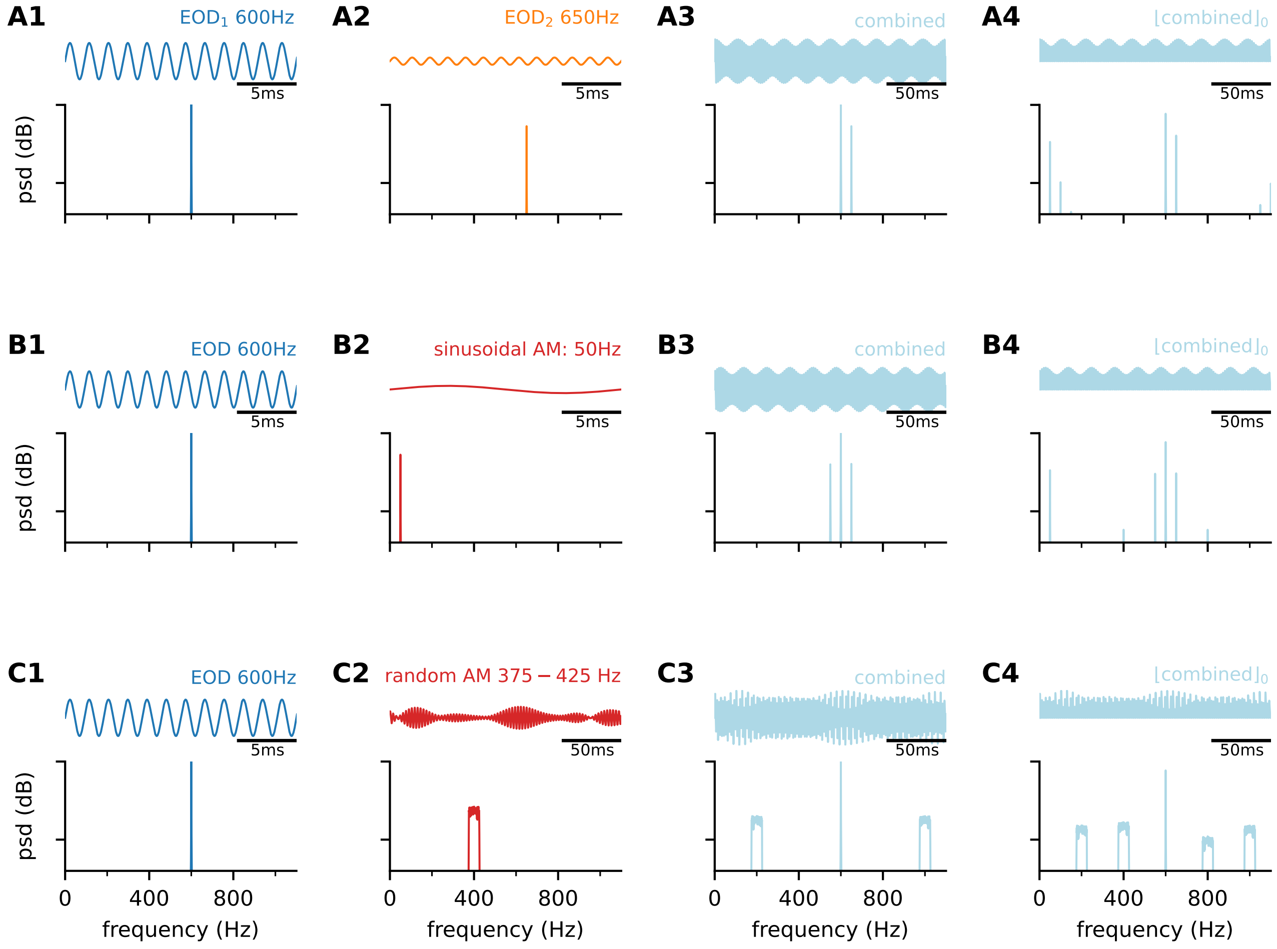

Figure 2. ***Direct*** **and** ***am*** **stimulation lead to stimuli with different spectral contents. A_1 – A_4** Direct stimuluation by adding a second signal (A_2) to the fish's self generated EOD (A_1). Relative amplitudes define the depth of resulting amplitude modulation of the combined signal (A_3, top). In the frequency domain, two peaks are observed at the individual frequencies. During sensory processing the combined signal undergoes a rectification which induces harmonics and a peak at difference frequency (50 Hz) arises. **B_1 – B_4** shows the am stimulation. The fish's EOD is combined with an amplitude modulated version of its own field. The desired sinusoidal AM is shown in B_2. B_3 and B_4 show the combined signal and the rectified version as in A. Note that the spectrum of the combined signal has the peak at the EOD frequency and two side-peaks. **C**1 – **C**$_4$ analogous but shows the combination with a random amplitude modulation as used for example by Savard et al. (2011).
